# The Use of Magnetic Resonance Imaging for Non-Invasive Assessment of Venofer® Biodistribution in Rats

**DOI:** 10.1007/s11095-018-2348-y

**Published:** 2018-03-08

**Authors:** Kimberley Span, Ebel H. E. Pieters, Wim E. Hennink, Annette van der Toorn, Vera Brinks, Rick M. Dijkhuizen, Geralda A. F. van Tilborg

**Affiliations:** 1 Department of Pharmaceutics, Utrecht Institute for Pharmaceutical Sciences (UIPS), Utrecht University,, Utrecht, The Netherlands; 2 Biomedical MR Imaging and Spectroscopy Group, Center for Image Sciences, University Medical Center Utrecht and Utrecht University, Utrecht, The Netherlands

**Keywords:** biodistribution, intravenous, iron, MRI, oxidative stress

## Abstract

**Purpose:**

The aim of this study was to determine the potential of magnetic resonance imaging to evaluate the biodistribution of exogenous iron within 24 h after one single injection of Venofer® (iron sucrose).

**Methods:**

Venofer® was evaluated *in vitro* for its ability to generate contrast in MR images. Subsequently, iron disposition was assessed in rats with MRI, *in vivo* up to 3 h and post mortem at 24 h after injection of Venofer®, at doses of 10- and 40 mg/kg body weight (*n* = 2 × 4), or saline (*n* = 4).

**Results:**

Within 10–20 min after injection of Venofer®, transverse relaxation rates (R_2_) clearly increased, representative of a local increase in iron concentration, in liver, spleen and kidney, including the kidney medulla and cortex. In liver and spleen R_2_ values remained elevated up to 3 h post injection, while the initial R_2_ increase in the kidney was followed by gradual decrease towards baseline levels. Bone marrow and muscle tissue did not show significant increases in R_2_ values. Whole-body post mortem MRI showed most prominent iron accumulation in the liver and spleen at 24 h post injection, which corroborated the *in vivo* results.

**Conclusions:**

MR imaging is a powerful imaging modality for non-invasive assessment of iron distribution in organs. It is recommended to use this whole-body imaging approach complementary to other techniques that allow quantification of iron disposition at a (sub)cellular level.

## Introduction

Iron is an essential nutrient for the transport of oxygen in the body. It is mainly present in heme, which is an important component of the oxygen-transporting protein hemoglobin that is present in erythrocytes. Insufficient intake and/or uptake can result in iron deficiency or anemia. In particular patients suffering from disorders such as chronic kidney disease (CDK) are in need of treatments such as erythropoietin and/or intravenous iron to enhance the number of erythrocytes (1–3). Several FDA-approved iron products are presently used in clinical practice, of which particularly iron sucrose has been widely used because this complex has shown to induce less side effects compared to other first generation intravenous iron complexes such as high molecular weight iron dextran (4,5). Although these iron complexes have been shown to be therapeutically beneficial for patients suffering from iron deficiency, there are some concerns that these iron products can also cause short- and long-term complications (6,7). After administration, the iron complexes which are colloidal dispersions of polynuclear ferric-oxyhydroxide cores stabilized by a carbohydrate shell, are taken up by phagocytosis by macrophages of the mononuclear phagocyte system (previously termed reticuloendothelial system (RES)) (8). In this respect, the colloidal iron particles follow the same fate of other nanoparticles based on for example synthetic polymers or lipids (9–12). In the macrophages, the complexes are degraded and the iron can be stored in ferritin or it can be released to bind to the blood plasma glycoprotein transferrin. The amount of iron released, relies on the physiological iron need and is speculated to also depend in part on the properties of the iron product, specifically related to the type of carbohydrate used to stabilize the iron complex (4,8,13). In case of iron overload, accumulation of iron in macrophages represents a safety concern as this might result in oxidative stress, inflammation, kidney- and heart disorders (6,14). Because of these safety concerns, there is a great need for insight into the distribution of iron after intravenous administration of iron-based medicinal products. In the reflection paper published by the European Medicine Agency concerning data requirements for the development of iron colloidal products for parenteral administration, a minimum requirement for biodistribution studies is outlined. This paper states that studies of iron distribution, retention and accumulation *in vivo*, should at least comprise analyzing the blood plasma, reticuloendothelial system and organ tissue (15). Furthermore, it is also desirable to examine the early iron distribution, meaning within twenty-four hours after iron administration, preferably in a non-invasive manner. Beshara *et al*. studied the long-term kinetics and distribution of iron after an intravenous injection, with a particular focus on iron distribution and the production of erythrocytes within 3–4 weeks after administration (16).

There is, however, no comprehensive knowledge available regarding the possible distribution of iron in the different organs within 24 h after administration. Giving insight into the iron distribution shortly after administration of the iron complexes elucidates on the early iron pharmacokinetics, which is useful when for example comparing two iron formulations. Several methods have been described to assess the distribution of iron complexes and quantitative disposition in different organs. Such methods mainly consisted of using radio isotopes, as for example ^52^Fe or ^59^Fe in positron emission tomography (PET) studies or using whole body counters (17–19). Previous studies using this approach to investigate iron disposition in several organs within a twenty-four-hour span after administration of the iron complexes, demonstrated that these methods allow qualitative assessment of iron biodistribution (16,18,19). Nevertheless, the use of radioactive labeling has its pitfalls such as safety and ethical issues and the need of excessive numbers of animals in the case of pre-clinical *in vivo* studies (20). Therefore, it is interesting to assess other methods to study the biodistribution of iron complexes. In a recent *in vivo* study, we investigated the toxicity effects and *in vivo* disposition of iron after intravenous administration by measuring iron parameters such as hemoglobin, serum iron and liver enzymes in blood serum, but also by performing histochemical studies to determine the tissue iron content and tissue inflammatory markers in rats. However, these parameters measured in the Sprague Dawley rat model did not give sufficient insight into the iron accumulation and adverse iron related effects after intravenous administration of different doses of iron products (manuscript submitted for publication).

Rostoker *et al*. (21) reported that it is feasible to study iron content in transfusion dependent patients suffering from iron overload or in hemochromatosis patients by using magnetic resonance imaging (MRI). In the past, the MRI technique has been used to investigate the delivery of iron nanoparticles such as liposomal SPION (superparamagnetic iron oxide nanoparticles) formulations (22). Thus, it would be of interest to investigate whether intravenously injected complex – bound iron can also be monitored with *in vivo* MRI. A study by Vinitski *et al*. reported that ferric hydroxide sucrose was successfully used as a MR contrast agent to image pulmonary embolism, suggesting the potential use of MRI for imaging of iron sucrose distribution (23). Iron has paramagnetic properties and can therefore reduce the magnetic resonance signal as the iron concentration increases (24).

Therefore, the aim of this study was to determine the potential of magnetic resonance imaging (MRI) to non-invasively and accurately monitor iron disposition within twenty four hours after one single injection of iron sucrose (Venofer®).

## Materials and Methods

### Materials

Venofer® (lot number: 118101) was obtained from Vifor (International) Ltd., St. Gallen, Switzerland. Saline (sodium chloride 0.9% *w*/*v*) was purchased from Fresenius Kabi Nederland B.V. (Zeist, The Netherlands). Heparin, 5000 U.I. (dissolved in water), was a product of Leo Pharma B.V, Amsterdam. Isoflurane was obtained from Teva Pharmachemie B.V., Haarlem, The Netherlands, and sodium pentobarbital 60 mg/ml in water, charge number 12042302, was provided by the pharmacy of Veterinary Medicine at Utrecht University, The Netherlands. Formalin solution neutral buffered 10% (HT501128-4 L), antibiotic antimycotic solution and sodium azide were purchased from Sigma-Aldrich Chemie B.V., Zwijndrecht, The Netherlands. Phosphate buffered saline (PBS) was from J.T. Baker (Avantor Performance materials B.V., Deventer, The Netherlands). Sarstedt S-monovettes tubes containing clotting activator silica beads for serum collection or EDTA for whole blood collection were purchased from Sarstedt B.V, Etten Leur, The Netherlands.

### Animals

All experiments were performed according to Institutional Ethical Committee Regulations of Utrecht University, The Netherlands.

#### MRI

Twelve male HSd: Sprague Dawley rats of 8 weeks old (Harlan laboratories, The Netherlands) were housed in standard perspex cages and were fed standard rat chow (Special Diets Services, United Kingdom) and (acidified) water *ad libitum* unless stated otherwise. The animals were housed for 4 weeks prior to the experiment and were subjected to a 12 h light/dark cycle. Each treatment group consisted of four animals per group (*n* = 4), weighing 325–400 g. The rats were randomly assigned to the different treatment groups, which were saline solution (control group) or Venofer® (20 mg (Fe)/ml) given in two different doses, namely: 10 mg (Fe)/kg and 40 mg (Fe)/kg. Each animal received one single intravenous bolus, instead of a drip infusion or slow injection as done in the clinic. The injection consisted of the volume Venofer® needed to reach 10 or 40 mg/kg, replenished with saline (sodium chloride 0.9% *w*/*v*) up to 1.5 ml.

#### Urine Iron Content Measurement

For the iron measurement in urine a total of eight Male HSd: Sprague Dawley rats weighing 325–400 g were used. The animals were divided in two treatment groups (*n* = 4 per group), specifically: the control group (saline solution) and 40 mg/kg of Venofer®. The experimental set-up for the measurement of iron content in urine is described in more detail further on in materials and methods in the section for iron measurement in urine.

#### Blood Half-Life

For the blood half-life experiment, eighteen male HSd: Sprague Dawley rats weighing 325–400 g were used. The animals were randomly assigned to different treatment and time-point groups for blood collection as described in more detail in the section; blood half-life of Venofer® in Sprague Dawley rats. Each animal received one single intravenous injection of (40 mg/kg) Venofer® or saline solution. The animals were housed and fed under the same conditions as described above.

### Blood Collection

In order to assess that the animals were healthy and non-anemic, 14 days prior to the MRI measurement blood was collected via the tail vein to measure the preteatment blood iron parameters. Rats were deprived from food for 19 h in order to avoid interference of iron present in food and had access to (acidified) water *ad libitum* before blood collection. The iron related blood parameters measured were: hemoglobin (HB), hematocrite (Hct), mean corpuscular volume (MCV), mean corpuscular hemoglobin (MCH), mean corpuscular hemoglobin concentration (MCHC), serum Fe (iron) and total iron binding capacity (TIBC) using an ADVIA 120 Siemens hematology analyzer and a Ferrentest (Diagnostic Chemicals Ltd., Charlottetown Canada).

### Iron Measurement in Urine after a Single Intravenous Injection

Animals received a single intravenous injection with a volume ranging from 650 to 750 μl conform to the weight of the rat, of Venofer® (40 mg/kg) or ~1.5 ml saline (control). Next, rats were placed in metabolic cages for 24 h, in which they had free access to (acidified) water *ad libitum* and 5 h to food. The aliment was then removed, resulting in a fasting period of 19 h. The urine of the different animals was collected for 24 h in a measuring cylinder containing 1 ml of antibiotic antimycotic solution diluted 1:5 with deionized water and the volume for each animal was recorded. Next, the urine was kept at −20°C until further assessment of the iron content which was determined using a Ferrentest (Diagnostic Chemicals Ltd., Charlottetown Canada).

### ***In Vitro*** Assessment of Venofer® as Contrast Agent for MRI

Venofer® was diluted in saline to obtain samples with different iron concentrations, ranging from 0.00125–0.04 M. MRI of the samples was conducted on a 4.7 T horizontal bore MR system (Varian, Palo Alto, CA, USA), using a home-built solenoid coil with a diameter of 3 cm. All measurements were performed at room temperature, using freshly prepared samples. T_1_ was measured using a Look Locker inversion recovery sequence (Total Repetition Time = 10 s, Repetition time per image = 25 ms, Echo Time (TE) = 4.2 ms, flip angle 5°, 100 images per inversion pulse, 2 averages, slice thickness 1 mm, field of view 30 mm × 30 mm, matrix 256 × 256). T_2_ values were assessed using a multi-slice multi-spin-echo (MEMS) sequence with the following parameters: Repetition Time (TR) = 3 s, TE = 8.52 ms, 100 echoes, 4 averages, slice thickness 1 mm, field of view 30 mm × 30 mm, matrix 256 × 256. T_2_^*^ values were obtained using a multi-slice multi-gradient-echo sequence (MGEMS) with the following settings TR = 500 ms, TE = 5 ms, 25 echoes, flip angle 70°, 16 averages, slice thickness 1 mm, field of view 30 mm × 30 mm, matrix 256 × 256. Average T_1_, T_2_ and T_2_^*^ values for each individual sample were established using non-linear fitting routines in Matlab. Next, relaxation rates R_1_ (1/T_1_), R_2_ (1/T_2_) and R_2_^*^ (1/T_2_^*^) were calculated, and used to determine relaxivities r_1_, r_2_ and r_2_^*^ based on the linear correlation between concentration and relaxation rate.

### Blood Half-Life of Venofer® in Sprague Dawley Rats

The blood half-life (t_1/2_) of Venofer® was obtained by MRI analysis of blood samples taken from the animals at several time points after a single intravenous injection of Venofer® as described above. In short, for each time point 2–3 Sprague Dawley rats received one single injection of Venofer® (40 mg/kg) or saline via the tail vein. Each rat was used to draw blood samples at two subsequent time points. For example, blood was collected from one rat at 30 min and 2 h post injection and the rat was then euthanized by an overdose pentobarbital administered intraperitoneally. Blood was collected in heparin coated Eppendorf tubes at time points 0 (pre-injection), and at 30 min, 1, 2, 3 and 24 h after injection of the formulation. The heparin coated Eppendorf tubes were prepared by pipetting 10 μl of heparin (5000 U.I) into 1.5 ml Eppendorf tubes and then allowing the heparin to concentrate overnight at 37°C. Next, blood was diluted 1:4 with saline and the samples were transferred into 250 μl PCR tubes for magnetic resonance imaging (MRI). Blood samples were stored at 4°C, and MRI was performed within 1–3 days after blood collection. MRI analysis of the blood samples was performed on a 9.4 T horizontal bore MR system (Varian, Palo Alto, CA, USA), using a Millipede™ coil (Varian Inc.). Shortly before the MRI measurements were carried out, the blood samples were vortexed for 30 s. T_2_-weighted images of blood samples were acquired, using a multi-slice multi-spin-echo sequence (MEMS) with the following parameters: TR = 5 s, TE = 9 ms, 200 echoes, 4 averages, field of view 30 mm × 30 mm, matrix size 256 × 256 and slice thickness 1 mm. Regions of interest were drawn within each tube, and average signal intensities at the different echo times were used to fit the average T_2_ value in each blood sample, using a non-linear fitting routine in Matlab. Subsequently, average T_2_ values were used to calculate the average relaxation rate R_2_ (1/T_2_) of each blood sample, as R_2_ scales linearly with contrast agent concentration. Next, blood half-life (t_1/2_) of Venofer® was obtained from the R_2_ values using a mono-exponential fitting routine in Matlab, according to the following function:

$$ {R}_2(t)={R}_2(0)\ast {e}^{\frac{-\ln (2)\ast t}{t_{\frac{1}{2}}}} $$

where R_2_ (t) is the relaxation rate at each time point, R_2_ (0) is the relaxation rate at time zero, t is time and t_1/2_ is the blood half-life (25). R_2_ values used for fitting were corrected for the average R_2_ value in blood samples from animals that did not receive any injections, resulting in a baseline R_2_ value of 0.

### Iron Disposition in Sprague Dawley Rats

#### *In Vivo* MRI of Iron Distribution after one Single Intravenous Venofer® Injection

Venofer® was intravenously administered via the tail vein to non-anemic Sprague Dawley rats. A total volume of 1.5 ml was administered consisting of a mixture of Venofer®, to obtain a dose of 10 or 40 mg/kg, supplemented with saline. As a control, 1.5 ml saline solution was injected into the rats. Shortly before MRI analysis, the animals were anesthetized with 3% isoflurane in air/O_2_ (1:2) and endotracheally intubated for mechanical ventilation. During MRI, the animals were mechanically ventilated and anesthetized with 2% isoflurane in air/O_2_ (4:1). Temperature, respiration, end-tidal CO_2_ levels, O_2_ saturation and heart rate were continuously monitored and kept within physiological range. *In vivo* MRI measurements were conducted on a 4.7 T horizontal bore MR system (Varian, Palo Alto, CA, USA), using a home-built Helmholtz volume coil with a diameter of 8 cm. Serial respiratory-triggered T_2_-weighted images of the rat abdomen were acquired over a period of 3.25 h using a multi-slice multi-spin-echo sequence (MEMS) with the following parameters: TR = 500 ms, TE = 4.1 ms, 9 echoes, 2 averages, field of view 64 mm × 64 mm, matrix size 128 × 128, 10 slices, 1 mm slice gap and slice thickness 1.5 mm. Total scan time per MEMS acquisition varied between 4.7 and 5.5 min for the different animals depending on the respiration rate. A total of 4 MEMS scans were acquired before intravenous injection of Venofer®, followed by approximately 3 h of repetitive MEMS acquisitions after injection. Following MRI, the animals were allowed to recover from anesthesia, transferred into normal cages and provided with *ad libitum* standard rat chow and (acidified) water until sacrificed 24 h after injection.

#### Postmortem MRI of Iron Distribution in Rats Euthanized at 24 h after One Single Intravenous Injection of Venofer®

For analysis of whole-body iron distribution at 24 h after a single intravenous injection of Venofer®, the rats described above in the *in vivo* MRI section, were prepared for postmortem MRI. Twenty four hours post injection, the rats were sacrificed using an overdose of pentobarbital that was administered intraperitoneally. Next, the animals were perfused with PBS followed by a 4% formaldehyde in water solution until they were completely blood depleted, and subsequently placed for further fixation in 4% formaldehyde for 7 days at 4°C. Subsequently, the formaldehyde solution was replaced by a PBS/0.1% azide solution, and the animals were stored at 4°C for postmortem MRI. Postmortem MRI measurements were conducted on a 4.7 T horizontal bore MR system (Varian, Palo Alto, CA, USA), using a home-built Helmholtz coil with a diameter of 8 cm. In order to cover the whole rat from head-to-toe, the animal was manually shifted by approximately 7 cm in between acquisitions, resulting in three acquisitions per rat. T_2_^*^-weighted images were acquired using a multi-gradient-echo 3D sequence (MGE3D) with the following parameters: TR = 100 ms, TE = 5 ms, flip angle = 25°, 8 echoes, 2 averages, field of view 100 mm × 90 mm × 60 mm and matrix size 250 × 226 × 150.

### MR Image Analysis ***In Vivo*** and Postmortem

For analysis of *in vivo* MRI data, regions of interest (ROIs) were manually outlined on the first MEMS acquisition of the time series in FSLView (FMRIB Software Library, (26,27). Next, ROI positioning was verified for the entire time series, and ROIs were manually adjusted to correct for motion. ROIs involved the liver, kidney, spleen, spine and muscle. Next, a non-linear fitting routine in Matlab was used to assess the average T_2_ value in each specific ROI at each individual time point. Venofer® -induced changes in R_2_ (1/T_2_) values, representative of Venofer® concentration, were used to evaluate Venofer® distribution. Afterwards, the area under the curve (AUC) for the different treatments in each organ was calculated, starting at baseline R_2_ at *t* = 0 until the end of the measurement of every individual rat, using GraphPad Prism version 4.02 for Windows. Subsequently, the average AUC of 4 rats within the different treatment groups was then calculated and statistical analysis was done to evaluate any differences *in vivo* between the treatments.

For analysis of postmortem MRI data, a predefined subset of organs, including the abdominal organs; liver, spleen, kidney, stomach and intestines and additional organs such as the heart, lungs, spine (bone marrow tissue), muscle, testis and brain, were manually outlined in the MGE3D images using FSLview. Next, a non-linear fitting routine in Matlab was used to assess the average T_2_* value in each specific organ. T_2_* values were used to calculate relaxation rates R_2_*, as described in the *in vitro* assessment of Venofer® section. In addition, the average signal intensity in the first T2*-weighted image (TE = 5 ms) of the MGE3D acquisition was calculated in the liver and spleen, and normalized to the average signal intensity in muscle tissue in the corresponding T2*-weighted image.

### Statistical Methods

The *in vivo* and postmortem data are expressed as mean ± standard deviation (st.dev). A two-way ANOVA with Bonferroni post-hoc testing was used to test the effects of the different Venofer® treatments on the Area Under the Curve from *in vivo* R_2_ values, postmortem R_2_* values and postmortem normalized signal intensity. Statistics was performed in GraphPad Prism version 4.02 for Windows, GraphPad Software, San Diego California USA. *P* < 0.05 was considered statistically significant.

## Results and Discussion

### Pretreatment Blood Collection

To assure that the rats were healthy and not anemic prior the MRI experiment, blood was collected via the tail vein 2 weeks prior to the start date of the experiment and several blood iron related parameters were measured (Table I).

**Table I:** Iron Related Blood Parameters

Sample (mg/kg)	HB (g/dL)	Hct (%)	MCV (fL)	MCH (pg)	MCHC (g/dL)	Serum Fe (μg/dL)	TIBC (μmol/L)
10	15.6	46	50.8	17.1	33.7	69.8	74.1
10	15.3	45	51.2	17.4	34.0	74.2	69.3
10	15.1	44	51.2	17.6	34.5	85.4	84.3
10	14.8	43	51.8	18.0	34.8	113.3	69.6
40	14.8	45	54.1	17.7	32.9	79.2	73.2
40	14.8	43	53.1	18.2	34.3	75.3	83.7
40	15.3	44	48.7	16.8	34.5	95.4	86.1
40	15.5	44	48.7	16.9	34.8	93.7	80.4
saline	15.0	43	52.8	18.4	34.6	153.5	77.1
saline	14.8	44	50.9	17.4	34.0	82.0	69.3
saline	15.5	45	51.1	18.4	34.8	87.6	80.4
saline	15.9	46	51.2	17.9	34.8	102.7	76.5

Iron related parameters for the individual rats in the different sample groups 14 days before the first injection

The parameter values shown in Table I were within normal physiological range of non- iron deficient rats as described in the manual for animal experiments, and as indicated by the animal supplier (28). The serum iron and total iron binding capacity values were in accordance with previously obtained results within our department (manuscript submitted for publication).

### Iron Measurement in Urine 24 h after a Single Intravenous Injection

**Fig. 1:**
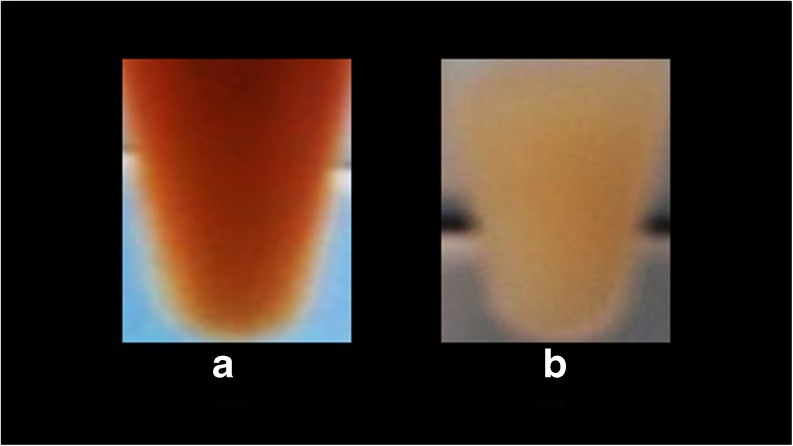
Photographs of urine collected 3 h (**a**) and 24 h (**b**) after one single intravenous injection of 40 mg/kg Venofer®.

Urine samples were collected in measuring cylinders for 24 h after one single Venofer® intravenous injection, as described in the section materials and methods for iron measurement in urine. A brownish color was observed in the urine collected at 3 h after administration in all rats receiving Venofer®, which was not observed in animals that received saline solution or in urine collected at 24 h post Venofer® injection (Fig. 1). This suggests that the exogenous iron is partly excreted via the urine within 24 h after administration.

In order to verify whether the brown color was indeed caused by iron, its concentration was measured in urine collected for twenty-four hours from animals receiving 40 mg/kg Venofer® or saline.

**Fig. 2:**
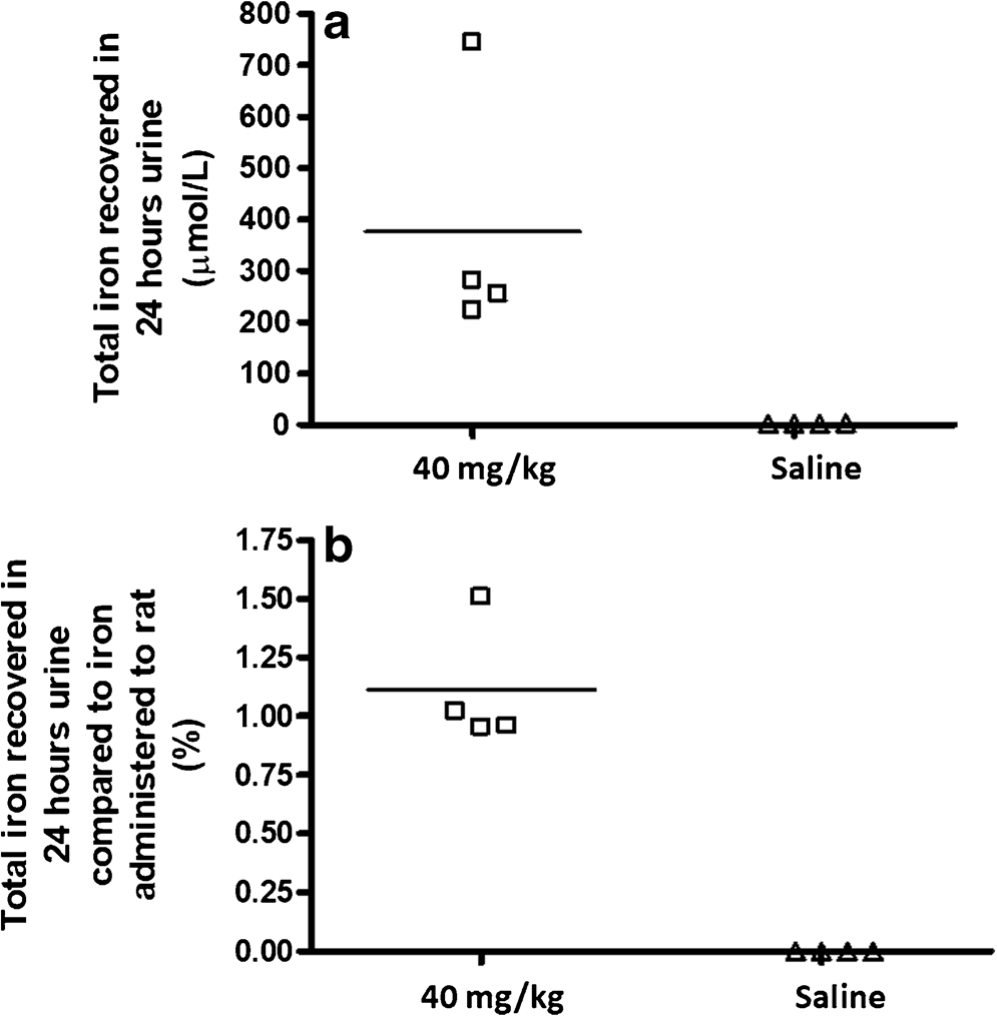
Total iron in urine collected for 24 h for each individual animal. Figure (**a**) depicts the concentration of iron in μmol/L and (**b**) shows the percentage of total amount of iron present in the urine compared to the administered iron dose per animal.

Figure 2 shows the total iron recovered in urine for the individual rats. The animals excreted an average of 20 ml urine in 24 h (range was 10–40 ml). For the rats that received saline solution, no iron was detected in the urine. This in contrast to the animals receiving 40 mg/kg Venofer®, which showed an average iron concentration of 380 ± 215 μmol/L. Urine-collected iron accounted for 1.1 ± 0.2% of the administered iron dose. This indicates that to some extent there is renal elimination of iron originating from the Venofer® injection. Danielson *et al*. also reported renal excretion in humans after receiving one intravenous Venofer® injection containing 100 mg Fe, 5% iron and 68% sucrose of the injected dose were excreted respectively after 4 h (29). A brown coloration of urine indicative of iron renal elimination, has also been reported for animals that received low molecular iron preparations (< MW 18 kD) (30). Venofer® has a molecular weight in the range of 34–60 kD and further research demonstrated that the average particle core size is smaller than 10 nm (31–33). Renal filtration is thought to be limited to 30–50 kD (around 5 nm) for proteins (34–36) making it plausible, that to some extent smaller iron complexes are filtered by the kidneys.

### ***In Vitro*** MRI Assessment of Venofer®

Venofer® was evaluated as a contrast agent for MRI prior to the *in vivo* experiment. Both the longitudinal (T_1_) and transverse (T_2_ or T_2_^*^) relaxation times for several Venofer® (iron) concentrations were measured to determine the relaxation rates (R_1_ = 1/T_1_ (s^−1^); R_2_ = 1/T_2_ (s^−1^) and R_2_^*^ = 1/T_2_^*^ (s^−1^)) as described in the materials and methods section for *in vitro* assessment of Venofer®. The slope obtained from linear regression analysis with iron concentration as the independent variable (x) and relaxation rate (R) as the dependent variable (y), provided the corresponding relaxivities r_1_, r_2_ and r_2_^*^, which give an indication of the efficiency of Venofer® to generate contrast on T_1_-weighted, T_2_-weighted or T_2_^*^-weighted magnetic resonance images, respectively.

**Fig. 3:**
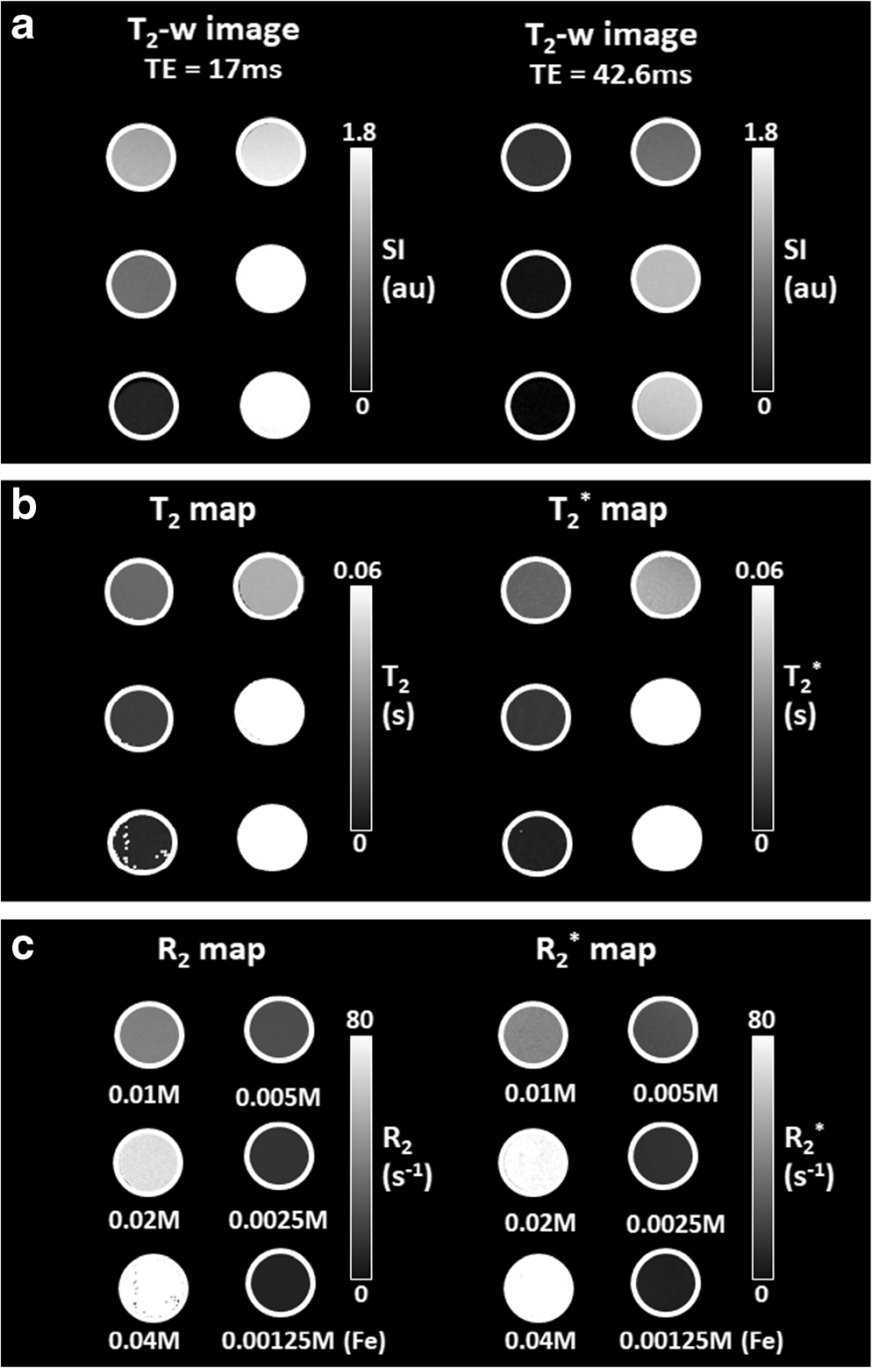
Effect of Venofer® at increasing iron concentrations on (**a**) T_2_-weighted images with two different echo times (TE), and its corresponding (**b**) T_2_ and T_2_^*^ maps and (**c**) R_2_ and R_2_^*^ maps. The concentrations are similar for Figures (**a**), (**b**) and (**c**).

The r_1_, r_2_ and r_2_^*^ relaxivities of Venofer® in saline solution at room temperature and 4.7 T magnetic field strength were respectively 0.45 mM^−1^s^−1^, 3.24 mM^−1^s^−1^ and 3.63 mM^−1^s^−1^ (Table II). These values indicate that Venofer® generates more efficient contrast on T_2_^(*)^-weighted magnetic resonance (MR) images than T_1_-weighted images. When compared to the relaxivities of a commonly clinically used T_1_ contrast agent at 4.7 T, such as Gadovist (Gd-DO3A-butrol), it can be concluded that Venofer® is less efficient as a contrast agent (relaxivities Gd-DO3A-butrol, r_1_ = 3.43 mM^−1^ s^−1^; r_2_ = 4.69 mM^−1^ s^−^1 (unpublished results). However, the iron complex does generate sufficient MRI contrast as it clearly generates a decrease in signal intensity on T_2_-weighted images, i.e. darker images, with increasing concentrations and increasing echo time (TE) as shown in Fig. 3a. This decrease in signal intensity is caused by the fact that iron shortens the intrinsic T_2_ relaxation time according to its paramagnetic properties (24,37). Quantitative T_2_- and T_2_^*^-maps are shown in Fig. 3b to visualize the effect of increasing iron concentrations on T_2_ and T_2_^*^ relaxation times. Furthermore, R_2_- and R_2_^*^-maps (1/T_2_ and 1/T_2_^*^) are shown in Fig. 3c to visualize the effect of increasing iron concentrations on R_2_ and R_2_^*^ relaxation rates. These maps clearly demonstrate that the highest concentration of Venofer® (0.04 M) most strongly reduced T_2_ and T_2_* values, whereas the lowest concentration (0.00125 M) had a smaller effect on T_2_ and T_2_* values. Moreover, these maps show that Venofer® affects T_2_ and T_2_^*^ relaxation times in a similar fashion, which was expected as relaxivities r_2_ and r_2_^*^ are comparable (Table II). Consequently, both T_2_- and T_2_^*^-weighted MR imaging sequences qualified for non-invasive monitoring of Venofer® biodistribution. *In vivo* experiments were performed using T_2_-weighted imaging (MEMS), whereas T_2_^*^-weighted imaging (MGE3D) was used for postmortem MRI.

**Table II:** Relaxivities

Contrast agent	r_1_ (mM^−1^s^−1^)	r_2_ (mM^−1^s^−1^)	${\mathrm{r}}_2^{\ast }$ (mM^−1^s^−1^)
Venofer®	0.45	3.24	3.63

Relaxivities r_1,_ r_2_, r_2_^*^ of Venofer® in saline solution at room temperature obtained from relaxation times measured at 4.7 Tesla magnetic field strength

### Blood Half-Life of Venofer® and ***In Vivo*** MRI of Iron Distribution

#### Blood Half-Life of Venofer®

The blood half-life (t_1/2_) of Venofer® was determined as described in the section materials and methods for blood half-life of Venofer® in Sprague Dawley rats. Here, T_2_ relaxation times of blood samples collected at 0, 30 min, 1, 2, 3 or 24 h after one single injection of 40 mg/kg Venofer® were measured at 9.4 T. The calculated blood half-life (t_1/2_) of Venofer®, based on a mono-exponential fit of the R_2_ (i.e. 1/ T_1/2_) values as a function of time, was 2.3 ± 0.6 h. In humans, a terminal blood half-life of t_1/2_ of 5.3 h was reported, based on the decay in iron concentration in the terminal phase (3–12 h post injection) (29). Longer elimination half-life, in humans compared to rodents, has also been reported for other types of nano-based medicines such as pegylated liposomal doxorubicin formulations (38).

#### *In Vivo* MRI of Iron Distribution

**Fig. 4:**
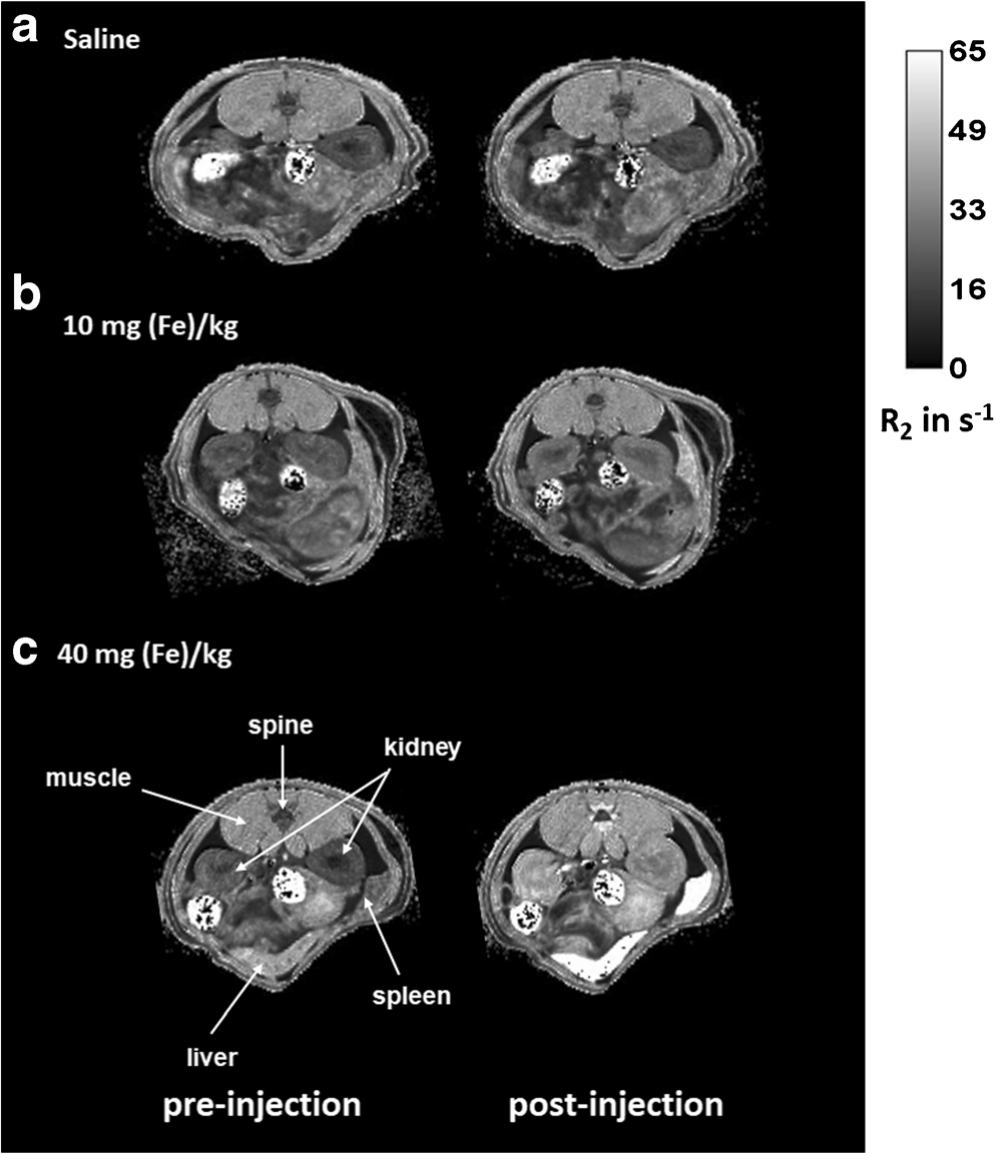
Representative examples of axial *in vivo* R_2_ maps of the rat abdomen depicting the organs pre- (left column) and post- (right column) intravenous injection of (**a**) saline; (**b**) 10 mg/kg Venofer® and (**c**) 40 mg/kg of Venofer®. Only one representative slice out of the 10 acquired slices is shown.

Axial multi-slice T_2_-weighted multi-spin-echo images were acquired to monitor iron distribution over different abdominal organs following Venofer® injection. Examples of resulting axial R_2_ (1/T_2_) maps of the abdominal region of animals pre- and post- one single injection of saline solution (control), 10 mg/kg or 40 mg/kg Venofer® are shown in Fig. 4. Changes observed in R_2_ values compared to pre-injection R_2_ values are proportional to the concentration of iron present in the organs, meaning that an increase of the relaxation rate R_2_ reflects more iron (see Fig. 3).

**Fig. 5:**
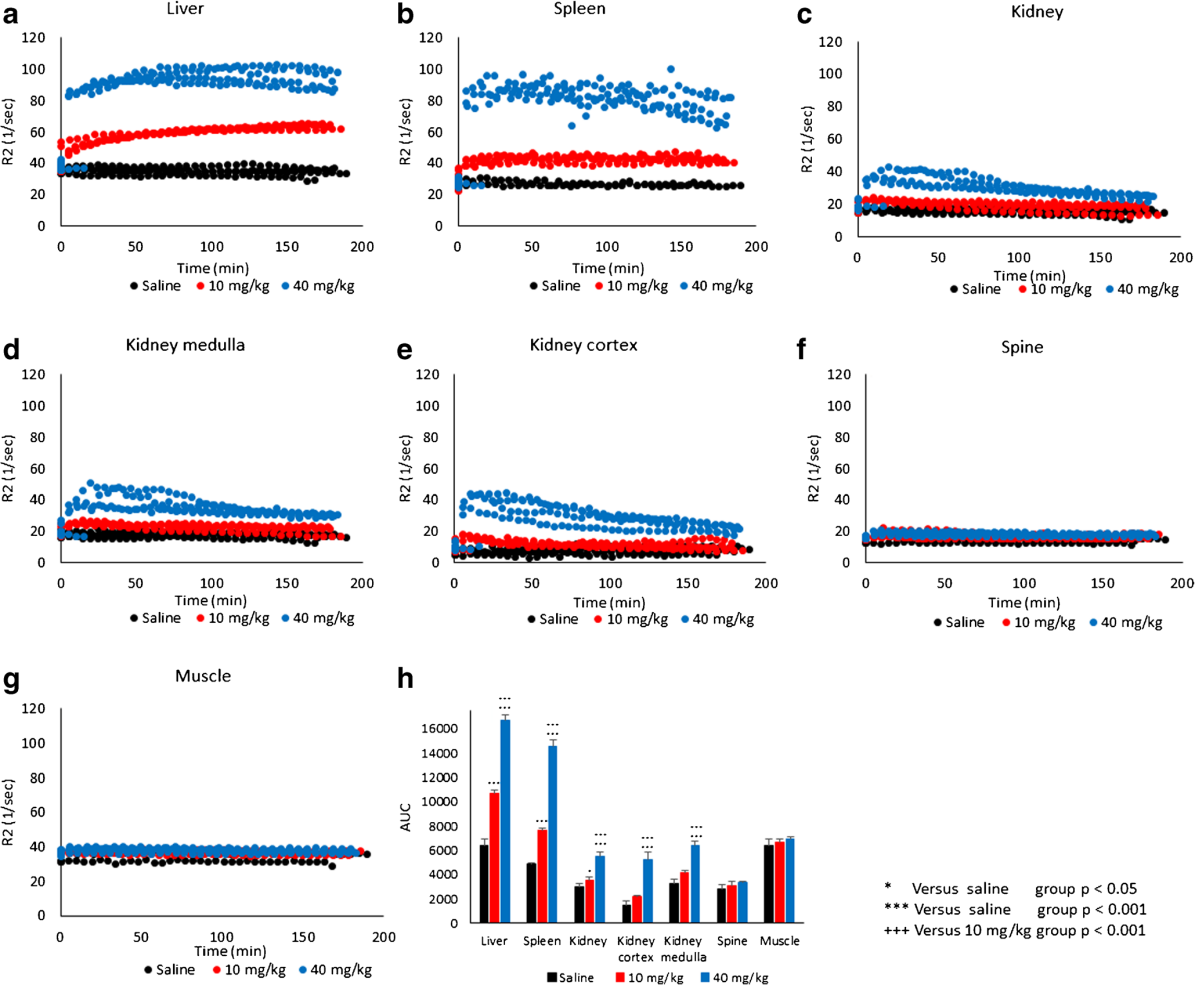
*In vivo* R_2_ values (s^−1^) of the different organs (**a**–**g**) as a function of time after one injection of saline (black), 10 mg/kg (red) or 40 mg/kg (blue) (*n* = 4 per group). Results are shown for each individual rat. R_2_ values at time zero are the average values of the 4 acquisitions that were acquired before injection for each individual rat. Figure (shows the area under the curve (AUC) for Figures (**a** to **g**). The results represent AUC mean ± st. dev of 4 rats.

For further quantitative assessment of the *in vivo* distribution of Venofer®, average R_2_ values were calculated in several regions of interest (ROIs), i.e. liver, spleen, kidney, spine (bone marrow) and muscle (Fig. 5).

In the liver (Fig. 5a), an injection of the frequently used dose for preclinical toxicity studies of 40 mg/kg, induced a rapid increase in R_2_ values that slowly continued to elevate reaching a plateau with an average R_2_ of 95 s^−1^ around 50 min post injection (57 s^−1^ higher compared to saline-treated control). Administration of 10 mg/kg of Venofer®, which is close to the maximum clinical dose of 7 mg/kg (39), also resulted in a fast increase of R_2_ values followed by a slow increase in R_2_ to reach maximum values of ~ 63 s^−1^ around 100 min after administration.

Similar to the liver, the spleen (Fig. 5b) demonstrated a steady increase in R_2_ values reaching maximum values at 30 min post injection that remained stable (low dose Venofer®) or showed a modest decrease (high dose Venofer®) towards the end of the *in vivo* measurements. Animals that received 40 mg/kg Venofer® showed an increase reaching maximum R_2_ values of 82 s^−1^ (54 s^−1^ higher than saline-treated control), while animals treated with 10 mg/kg Venofer® reached maximum R_2_ values of 43 s^−1^ (14 s^−1^ higher than saline-treated control).

The kidney also showed a rapid increment in R_2_ after administration of 40 mg/kg Venofer®. However, unlike the liver and spleen, the initial increase in R_2_ was followed by a more obvious gradual decrease over time (Fig. 5c). Three hours post-injection, R_2_ values of the whole kidney, as well as for the separate compartments (Fig. 5d, e; cortex and medulla), decreased to values not different from the pretreatment or saline values both at high and low dose Venofer®. In addition, there was no distinction between the two sections of the kidney (cortex and medulla) during the *in vivo* measurements at either dose. Compared to all other regions of interest, spine, which served as a measure for bone marrow, and muscle tissues showed only modest changes in R_2_ after injection with the iron complex (Fig. 5f, g). Figure 5h depicts the area under the curve (AUC) for the R_2_ values over time in each organ. The AUC in the liver, spleen and kidney (including both the compartments cortex and medulla) of animals that received 40 mg/kg Venofer®, was significantly increased compared to animals in the 10 mg/kg and saline (control) treated groups (*p* < 0.001). Furthermore, also the 10 mg/kg Venofer® dose, which is comparable to the maximum clinical dose, significantly increased the AUC compared to saline-treated control animals in liver, spleen (p < 0.001) and kidney (*p* < 0.05).

This clearly demonstrates the potential of MRI to measure the *in vivo* distribution of the iron sucrose complex at relatively high dose.

Absolute concentrations of deposited iron in the organs of interest cannot be quantified because the r_2_ relaxivity of Venofer®, as assessed *in vitro*, may change *in vivo*. For instance due to release of iron from the sucrose complex or due to internalization of the iron complex by cells of the RES (8). Moreover, changes in R_2_ values do not only originate from deposited iron, but may partly originate from Venofer® that is circulating in the blood, particularly in highly vascularized tissues such as spleen and liver. The blood half-life of Venofer® in rats is 2.3 ± 0.6 h, meaning that the contribution of vascular Venofer® to the measured R_2_ values should gradually decrease over the time course of the longitudinal *in vivo* MRI experiment, and only around 40% of the injected dose will be present in the blood at the final acquisition 3 h post-injection. Nevertheless, R_2_ values remained constantly elevated over the time course of 3 h in the liver and spleen, indicating that these organs are the main organs of exogenous iron disposition after a single injection of the iron complex.

### Post Mortem MRI of Iron Distribution in Rats Euthanized 24 h after One Single Injection of Venofer®

**Fig. 6:**
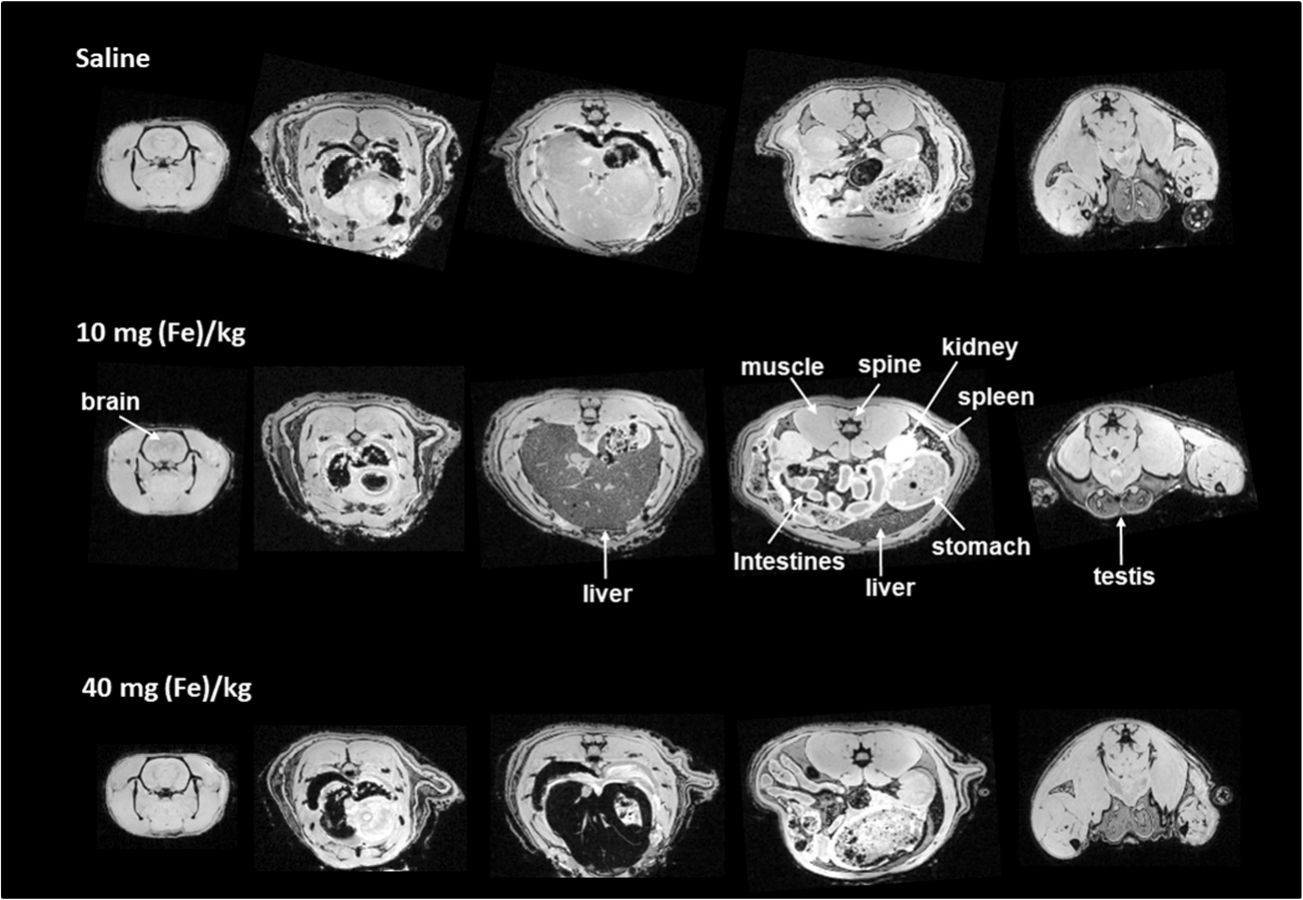
Representative T2^*^-weighted images (TE = 5 ms) of the whole rat, from head (left) to toe (right), after administration of saline, 10 - or 40 mg/kg Venofer®.

**Fig. 7:**
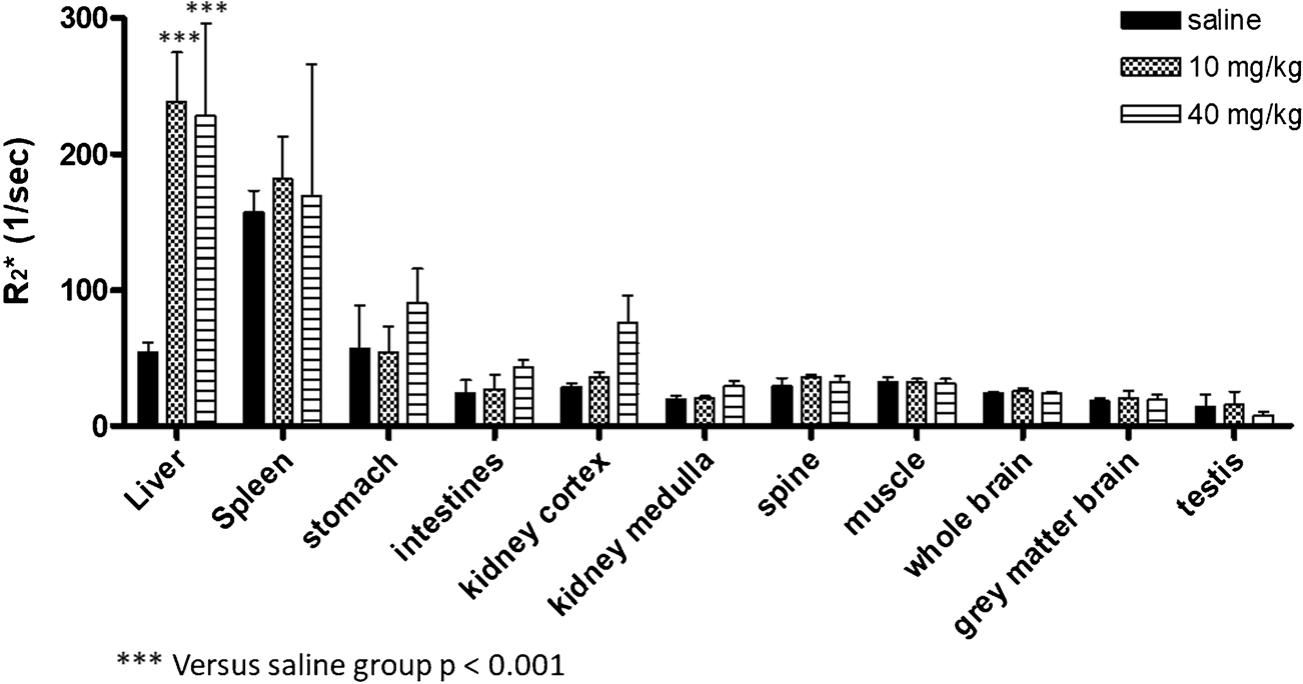
Average R_2_^*^ values (s^−1^) for different organs measured post mortem in rats euthanized 24 h post injection of 10 - or 40 mg/kg Venofer® or saline solution (*n* = 4 per group). Results represent mean ± st.dev for 4 rats.

Postmortem T_2_*-based MRI (multi-gradient-echo 3D (MGE3D)) was performed to evaluate whole-body iron disposition, 24 h post Venofer® injection. Three postmortem imaging sessions were performed for each rat to allow whole-body coverage with an 8 cm diameter Helmholtz volume coil (Fig. 6), and ROIs were manually drawn in different organs of interest to calculate mean R_2_* values from the MGE3D images (Fig. 7).

ROI-based analysis in rats that received Venofer® showed increased R_2_^*^ values compared to saline-treated control animals, indicative of iron uptake in the liver (Fig. 7). R_2_^*^ values from organs such as the lungs and the heart, which are also considered target organs for iron accumulation, are not depicted in Fig. 7 as these values may be affected by the presence of air and should therefore be treated with care.

**Fig. 8:**
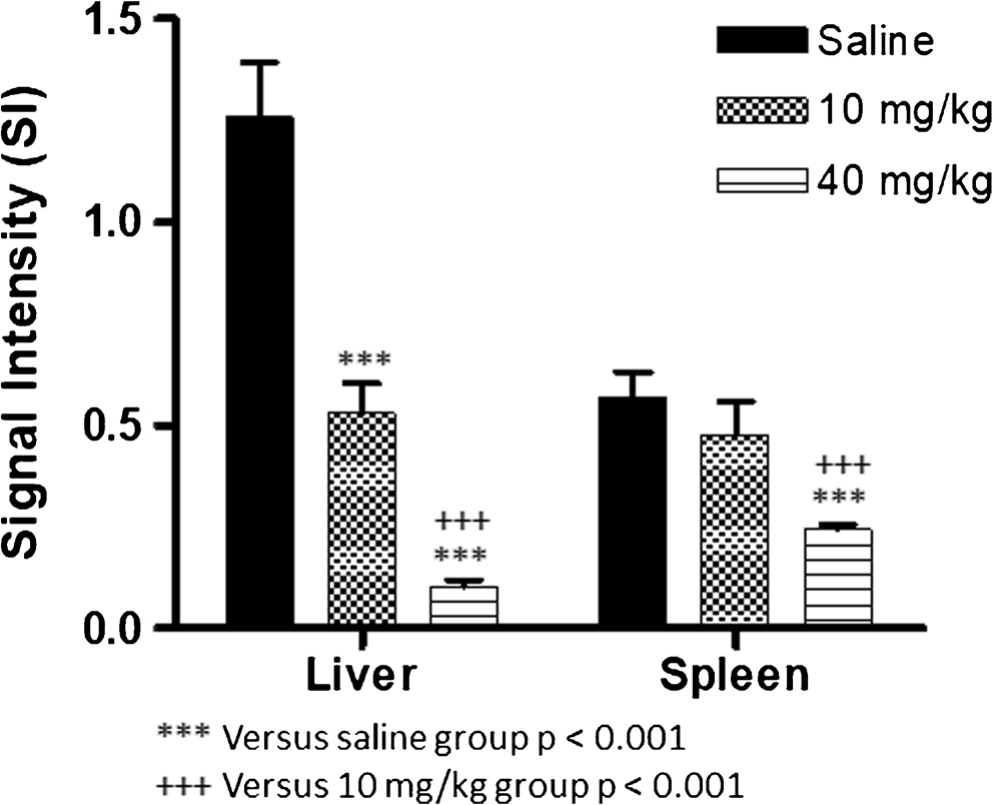
Average signal intensity in the liver and spleen, normalized to the signal intensity in muscle tissue in the corresponding MR acquisition. Results represent mean ± st.dev for 4 rats.

The observed R_2_^*^ values in the liver and spleen suggest that there is no significant difference in iron accumulation between animals treated with 10 or 40 mg/kg (Fig. 7). However, this outcome is not in accordance with Fig. 6, where a clear decrease in signal intensity was observed in these organs with increasing dose from 10 to 40 mg/kg. This discrepancy may be explained by substantial iron accumulation in these organs, resulting in T_2_^*^ values that were too low for accurate T_2_^*^ quantification. Therefore iron accumulation in the liver and spleen was also studied by quantification of the average signal intensity in these organs in the T_2_^*^-weighted images (TE = 5 ms), after normalization to the average signal intensity of muscle tissue in the corresponding images (Fig. 8). Both in liver and spleen, the normalized signal intensity was significantly lower in the animals that received the 40 mg/kg dose as compared to the saline-treated controls (Fig. 8). Moreover, both organs demonstrated a significant decrease in signal intensity with increasing Venofer® dose.

The outcomes of this MRI experiment demonstrate that within 24 h of one single administration of two different doses of Venofer®, the liver and spleen are the main organs for exogenous iron disposition. This coincides with previous reports describing the most adopted mechanism, in which intravenously injected iron complexes are taken up by the mono nuclear phagocyte system and engulfed by macrophages to next dissociate the iron from the complex. Subsequently, the iron is then presumably mainly absorbed by the liver, spleen and bone marrow. (8,13).

Besides chemical and physical characterization of the iron nano-colloidal complexes, a biodistribution study is necessary in order to perform a proper comparison between different iron products. PET imaging with radioisotope-labeled iron and inductively coupled plasma mass spectrometry (ICP-MS) techniques have been demonstrated to allow assessment of iron content in different organs (3,16). Nevertheless, an MRI-based method that allows longitudinal non-invasive monitoring of iron distribution is considered more advantageous, as this imaging modality does not require modifications of the iron complex with radioactive labels, as needed for PET. Moreover, there is no need to sacrifice animals at several time points such as in the case of ICP-MS measurements, and it is hence expected to be easily translated to the clinic.

The present study shows that T_2_-based MRI allows continuous and non-invasive monitoring of iron distribution over various organs of interest. Despite the obvious advantages, there are some limitations to the present method. First, iron complexes such as Venofer® induce relatively low MRI contrast, which limits its detection to relatively high iron concentrations. It should also be taken into account that due to differences between the various iron complexes, each iron product should be separately evaluated for their ability to generate sufficient MR contrast. Second, the spatial resolution of MRI only allows monitoring iron disposition at macroscopic level but cannot be used when interested in cellular iron disposition which can be studied with microscopy techniques (40). Third, R_2_^*^ changes in organs cannot be directly converted into iron concentrations, as intracellular iron accumulation may alter its relaxivities (24,41).

Therefore, in order to scrutinize differences in biodistribution and fate of the various iron preparations as proposed by the European Medicines Agency (EMA) (15), a multimodal study should be carried out. Several evaluation techniques have to be combined to encompass the full scope of iron accumulation in plasma, mononuclear phagocyte system and organ tissues, after administration of iron-based medicines.

## Conclusion

MRI was successfully used for non-invasive monitoring of iron distribution up to twenty-four hours after one single intravenous injection of Venofer® in rats. The present method is considered complementary to existing iron quantification techniques, and could therefore be a valuable whole-body imaging approach for biodistribution measurements to compare iron-based medicines in preclinical studies.

## Abbreviations

EMA: European medicine agency
HB: Hemoglobin
Hct: Hematocrite
ICP-MS: Plasma mass spectrometry
MCH: Mean corpuscular hemoglobin
MCHC: Mean corpuscular hemoglobin concentration
MCV: Mean corpuscular volume
MEMS: Multi-slice multi-spin-echo sequence
MGE3D: Multi-gradient-echo 3D
MGEMS: Multi-slice multi-gradient-echo sequence
MRI: Magnetic resonance imaging
PET: Positron emisssion tomography
r_1_, r_2_, r_2_*: Relaxivities
R_2_: Transverse relaxation rate
ROIs: Region of interest
Serum Fe: Serum iron
SPIONs: Superparamagnetic iron oxide nanoparticles
T_1_: Longitudinal relaxation time
t_1/2_: Blood half-life
T_2_: Transverse relaxation time
TE: Echo time
TIBC: Total iron binding capacity
TR: Repetition time
